# Effects of MicroRNA on Regulatory T Cells and Implications for Adoptive Cellular Therapy to Ameliorate Graft-versus-Host Disease

**DOI:** 10.3389/fimmu.2018.00057

**Published:** 2018-01-31

**Authors:** Keli L. Hippen, Michael Loschi, Jemma Nicholls, Kelli P. A. MacDonald, Bruce R. Blazar

**Affiliations:** ^1^Department of Pediatrics, Division of Blood and Marrow Transplantation, University of Minnesota Cancer Center, Minneapolis, MN, United States; ^2^The Antigen Presentation and Immunoregulation Laboratory and Bone Marrow Transplantation Laboratory, QIMR Berghofer Medical Research Institute, University of Minnesota Cancer Center, Brisbane, QLD, Australia

**Keywords:** regulatory T cell, tTreg, iTreg, microRNA, graft-versus-host disease

## Abstract

Regulatory T cells (Tregs) are key mediators of the immune system. MicroRNAs (miRNAs) are a family of ~22 nucleotide non-coding RNAs that are processed from longer precursors by the RNases Drosha and Dicer. miRNA regulates protein expression posttranscriptionally through mRNA destabilization or translational silencing. A critical role for miRNA in Treg function was initially discovered when both Dicer and Drosha knockout (KO) mice were found to develop a fatal autoimmune disease phenotypically similar to Foxp3 KO mice.

## Introduction

Regulatory T cells (Tregs), CD4+ 25+ Foxp3+ cells, are key mediators of the immune system, which function to suppress self-reactive lymphocytes (1) and limit immune responses to chronic pathogens and commensal bacteria (2). Treg development and suppressive function are tightly regulated. Genetic mutations that prevent Treg lineage specification lead to severe autoimmune diseases, whereas expanded Treg numbers lead to global immunosuppression and inhibit the clearance of tumors and opportunistic infections (3, 4). Allogeneic hematopoietic stem cell transplantation (HSCT) is a curative option for many hematological malignancies. Graft-versus-host disease (GVHD) (5) occurs in 40–70% of recipients, with skin, liver, and gut representing major GVHD target organs (6). Adoptive Treg transfer is effective at preventing autoimmunity, organ rejection, and GVHD in preclinical models (1, 5), and Treg therapy reduces disease in human clinical trials in GVHD and stabilized C-peptide levels for >2 years in several individuals with diabetes (7–10).

MicroRNAs (miRNAs) are a family of ~22 nucleotide non-coding RNAs that contain a short seed region complementary to mRNAs (typically the 3′ UTR) sequences. miRNA regulates gene expression posttranscriptionally through repression of protein production by mRNA destabilization or translational silencing. miRNAs are produced as longer primary transcripts (pri-miRNAs) by RNA polymerase II or III. pri-miRNAs are processed into mature miRNAs by the RNases Drosha and Dicer and are incorporated into the RNA-induced silencing complex. miRNAs instruct the differentiation, suppressive function, and stability of thymically derived Treg (tTreg) and Treg induced in the periphery (pTreg) or *in vitro* (iTreg) (11–14). Tregs exhibit a distinct miRNA profile compared with conventional T cells (15), and individual miRNA or miRNA clusters contribute to Treg biology through distinct mechanisms. The expression of the Treg defining transcription factor Foxp3 itself shapes the Treg miRNA profile. Moreover, miRNAs promote Foxp3 expression during iTreg generation. Finally, it is now widely recognized that expression of Foxp3 does not endow a terminal state of differentiation, and Tregs have a degree of plasticity, and miRNAs are required to integrate the external signals that drive this phenomena (16). This review focuses on how our knowledge of Treg pathways controlled by miRNA can be applied to improve the efficacy of Treg cellular therapy, with an emphasis on GVHD.

### miRNA Effects on Thymic Treg (tTreg) Differentiation

In mouse models (17–20), the deletion of miRNAs by lineage-specific ablation of Dicer or Drosha in T cells or Treg specifically reduces the number of tTreg and pTreg precipitating fatal multiorgan inflammatory disease. Cell autonomous miRNAs are required for tTreg development in the thymus and Foxp3 induction by TGF-β during iTreg generation (19). These results have been confirmed (21) using Tie2Cre- and CD4Cre-mediated Dicer deletion mouse models where miRNA depletion in either hematopoietic/endothelial cells or thymocytes leads to a twofold to threefold decrease in Foxp3+ Treg frequencies.

**Figure 1:**
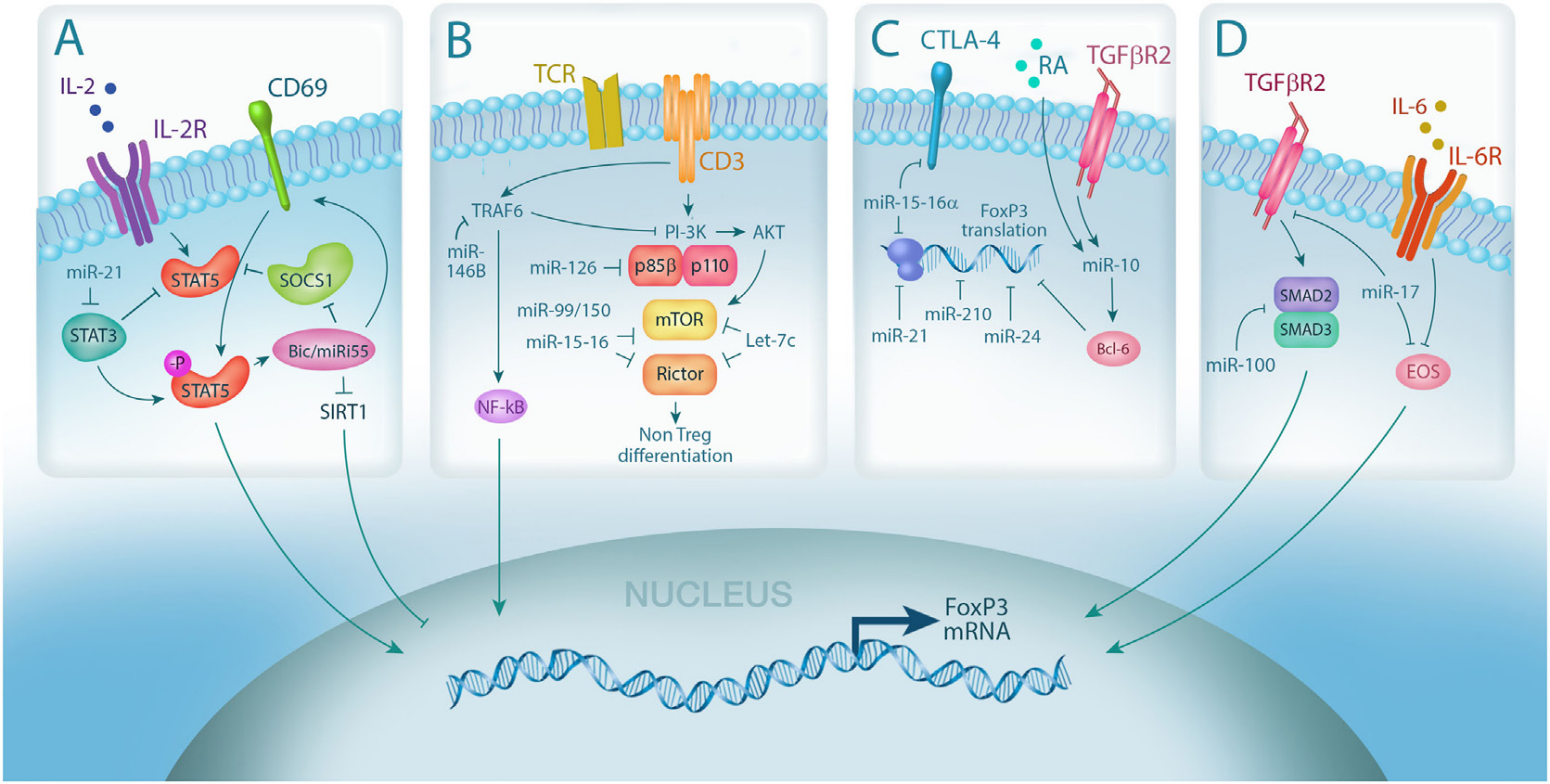
MicroRNA (miRNA) integrates multiple pathways in regulatory T cell (Treg). **(A)** IL-2 signaling is required for Treg differentiation and is enhanced by miRNA-mediated downregulation of the IL-2R signaling inhibitors, suppressor of cytokine signaling 1 (SOCS1) (miR-155) and STAT3 (miR-21); **(B)** miRNA expression controls Treg induction by negatively regulating TCR signal strength *via* downmodulation of PI3Kβ (miR-126) and mammalian target of rapamycin (mTor)/Rictor (miR-99/150, miR-15-16, and Let-7); **(C)** Foxp3 mRNA stability and translation can also be directly negatively regulated by miRNA *via* (miR-15-16a, miR-24, and miR-210) or positively regulated through indirect mechanisms (miR-10, *via* Bcl-6); **(D)** miRNA also controls Treg plasticity by regulating the expression of transcription factors required for Foxp3 transcription, including SMADs (miR-100) and EOS (miR-17).

miR-155, controlled by Dicer, is regulated by Foxp3 that binds the host gene *bic* promoter region (19, 22–24). Murine bic/miR-155 deficiency resulted in reduced thymic and splenic Treg as a consequence of impaired tTreg development but did not alter Treg function or *in vivo* homeostatic proliferation (Table 1) (25). However, as discussed below, miR-155 knockout (KO) Tregs have inferior Foxp3 expression and stability as well as fitness compared with wild type Tregs (24) but without defective *in vitro* TGF-β-mediated Foxp3 induction in CD4+ T cells (25). miR-155 KO mice have a reduced proportion and absolute number of Foxp3 cells, accompanied by diminished STAT5 signaling, downstream of the IL-2R complex, and higher suppressor of cytokine signaling 1 (SOCS1), a negative regulator of STAT5 signaling (24). Indeed, miR-155 effects are partially mediated by targeting SOCS1, a negative regulator of the IL-2 signaling pathway with a crucial role in Treg development (26). In a positive feedback loop, it has been suggested that during tTreg differentiation, induction and upregulation of Foxp3 expression reciprocally drive high expression of miRNA-155 (24). Similarly, the C-type lectin receptor, CD69, was found to control tTreg development, peripheral Treg homeostasis, and iTreg generation *via* STAT5 signaling effects; miR-155 induced CD69 (27). Similarly, in human, miR-155 (and miR-124a) was shown to repress the histone deacetylase, sirtuin-1, resulting in higher Foxp3 expression and iTreg generation (28). Strategies to augment miR-155 and miR-124a or conversely to downregulate SOCS1 should increase STAT5 signaling and Treg responses to IL-2 (Figure 1A).

**Table 1:** Role of microRNA (miRNA) in regulatory T cell (Treg) induction, Foxp3 expression, and suppressive function.

miRNA	Effect on development	Mediated by	Reference
**miRNA involved in tTreg development**			
miR-155	Increases expression	Suppressor of cytokine signaling 1 (SOCS1)	(61)
miR-146a	Decreased	STAT1	(54)
miR-146b	Decreases expression	TRAF6/NF-κB	(65)
**miRNA involved in iTreg development**			
miR-15b/16	Increased	Mammalian target of rapamycin (mTor)/Rictor	(30)
miR-99a	Increased	mTor	(29, 47)
miR-126	Increased	p85β	(32)
miR-150	Increased	mTor	(31)
miR-155	Increased	SOCS1	(61)
miR-17	Decreased	TGFβRII	(40)
miR-100	Decreased	SMAD2/3	(31)
**miRNA controlling Foxp3 mRNA stability/translation**			
miR-15a-16	Decrease	Direct effect	(48)
miR-24	Decrease	Direct effect	(47)
miR-31	Decrease	Direct effect	(36)
miR-210	Decrease	Direct effect	(47)
miR-10a	Increase	Bcl-6	(38, 45)
miR-95	Increase	Unknown	(47)
**miRNA effects on suppression-related molecules**			
miR-15a-16	Decreases suppression	CTLA-4	(48)
let-7d	Increases suppression	Exosomes	(67, 72)
miR-155	Increases Teff susceptibility to Treg	Unknown	(73)

### miRNAs Effect on the Differentiation of *In Vitro* Induced Treg (iTreg)

Dicer also regulates iTreg generation and differentiation (Figure 1B) (19). *In vitro* TGF-β induces Foxp3 less efficiently in naïve Dicer-deficient CD4+ T-cells compared with wild type counterpart with similar results observed in mice harboring CD4-restricted Drosha or Dicer deficiencies (17). With Drosha or Dicer deficiency, the observed decreased Foxp3 expression after TGF-β stimulation could not be overcome by retinoic acid (RA) addition. These studies provided a rationale for an extensive miR screen, which lead to the identification of positive regulators of iTreg generation (29). miR-10b, miR-99a, miR-130a, miR-146b, miR-150, and miR-320 were amongst those found to drive Treg differentiation. With the multitude of miRNAs revealed by the screening, it was proposed that an miRNA network, rather than individual miRNAs, controls iTreg differentiation. For example, miR-99a cooperates with miR-150 to repress the expression of the mammalian target of rapamycin (*mTor*), a known inhibitor of iTreg differentiation (29). miR-150 antagomir exposure led to a reduced iTreg differentiation. Whereas miR-99a expression was upregulated by RA exposure and repressed *mTor* by binding to the 3′ UTR (29), miR-150 only repressed *mTor* in the presence of miR-99a (29). Similarly, miR-15b-16 (30) and miR-15a-16 function to reduce *mTor* signaling. miRNA-dependent *mTor* pathway downregulation supports human iTreg generation (31) as evidenced by inhibition of iTreg differentiation in CD4+ T-cells overexpressing miR-100. The specific miR-100 editing (C-to-U transversion) changes the miR-100 target from *mTor* to *SMAD2*, augmenting TGF-β signaling and iTreg differentiation. *mTor* and the PI3K–AKT pathways were identified as miR-126 targets in both mouse and human Treg (32). miR-126 inhibits PI3K p85β, responsible for AKT upregulation, and in so doing, augments Foxp3 and iTreg differentiation. Conversely, miRNA-126 silencing reduced iTreg generation and Foxp3 expression *via* enhanced p85β, pAKT, and mTOR, consistent with a critical effect of PI3K/Akt pathway on Treg Foxp3 expression (33, 34). As in tTreg generation, miR-155 facilitates iTreg generation (35) by downregulating SOCS1 and hence promoting JAK/STAT signaling (26).

Not all miRNAs have a positive effect on iTreg differentiation. miR-31 and the miR-17–miR-92 cluster function as negative regulators of iTreg differentiation (29). miR-31 represses human Treg Foxp3 expression (36) while the miR-17–miR-92 cluster represses iTreg formation (37–40). miR-17 directly targets TGF-β receptor II and the cAMP-responsive element binding protein 1, both implicated in bolstering Treg differentiation. It has been proposed that strong CD28 signals inhibit Foxp3 induction, which may be influenced by costimulatory signaling pathways that induce miR-17–miR-92 (41). Similarly, miR-23–miR-27–miR-24 cluster overexpression impairs TGF-β-mediated Treg induction (42). Together, these data identify miRNA agonist targets (miR-99a, miR-150, iR-15b-16, miR-100, miR-126, and miR-155) that can be exploited to increase iTreg generation. Moreover, miRNA (miR-31, miR-17–miR-92, and miR-23–miR-27–miR-24) antagomir treatment of T cells *in vitro* may be exploited to support iTreg generation, while *in vivo* treatment may foster pTreg generation.

### miRNA Effects on Foxp3 Expression

miR-10a, which is induced in Treg following RA and TGF-β exposure, is one of few miRNAs exclusively expressed in tTreg (38, 43, 44). Although not required for Foxp3 expression, miR-10a contributes to Treg stability by targeting the transcriptional repressor Bcl-6 resulting in high and sustained Foxp3 (Figure 1C) (38, 43, 45). In this regard, miR-10a antagomir-treated Tregs exhibit decreased Foxp3 protein expression (46). In addition, miR-95 is also highly expressed in human Treg and, through an unknown mechanism, enhances Foxp3 mRNA and protein expression (47).

By contrast, miRNA can also function as negative regulators of Foxp3 expression (47, 48). Foxp3 is a direct target of miR-15a-16, which is expressed at low levels in human Treg (40). Forced overexpression of this miRNA in Treg markedly reduced Foxp3 and CTLA-4 expression, concordant with reduced suppressor function, whereas miR-15a-16 deletion in conventional T-cells upregulated Foxp3 and CTLA-4 expression (40). Similarly, forced expression of either miR-24 or miR-210 in Treg resulted in a twofold decrease in Foxp3 expression demonstrating Foxp3 as a direct target of these miRNAs (47). Thus, to increase Foxp3 expression and promote Treg stability and suppressor function, approaches can be undertaken to increase miR-10a or miR-95 and/or decrease miR-15a-17, miR-24, or miR-210.

### miRNA Regulation of Treg Stability

As briefly discussed earlier, Tregs exhibit some degree of plasticity, especially with the Th17 subset of CD4+ T cells whose differentiation, like inducible Treg, is driven by TGF-β (49). miRNAs also have been shown to be critical in the maintenance of Treg stability that is required for the preservation of suppressor function during inflammation that can occur as a result of GVHD and autoimmune disease. Thus, the following miRNAs present potential new targets for the regulation of Treg stability *in vivo*.

miR-21, a key regulator of Treg stability (15, 36, 50), is more highly expressed in Treg compared with conventional T-cells and regulates Foxp3 expression and Treg proliferation (36, 50). A significant reduction in miR-21 and Foxp3 mRNA was noted in tTreg from rheumatoid arthritis patients compared with healthy controls (50) and was accompanied by increased STAT3, decreased STAT5 protein expression, and skewing toward Th17 cells. These data led to the hypothesis that miR-21 is part of a negative feedback loop dysregulated during disease that contributes to Th17 and Treg imbalance in rheumatoid arthritis patients (50). Evidence supports that miR-21 regulates Foxp3 expression and Treg homeostasis by modulating STAT3 and STAT5 (36, 50). Peptide nucleic acid (PNA) inhibition was used to block miR-21 expression to delineate its effects on human Treg functions (15). Although PNA-inhibited Treg failed to proliferate under anti-CD3 mAb stimulation conditions (15), suppression was unchanged, supporting the contention that miR-21 expression promotes Treg stability and homeostasis.

Foxp3 regulates gene expression by recruiting corepressors and coactivators, and miRNA directly regulates Treg function by controlling the expression of these cofactors. For example, IL-6, which is an established mediator of acute GVHD in mice and patients (51) and required (with TGF-β) to induce Th17 cells, induces the expression of miR-17. miR-17 targets Eos, a transcription factor that cooperates with Foxp3 to mediate suppressor gene expression (Figure 1D) (52). Decreased Eos expression causes derepression of effector cytokine genes and exuberant cytokine responsiveness. In line with this, transgenic miR-17 overexpression exacerbates pathology in a murine colitis model (52). Conversely, miR-146a targets STAT1 expression and minimizes IFNγ/STAT1-mediated loss of Treg-suppressive function. This plasticity is mediated by T-bet, the Th1-specifying transcription factor, which promotes Treg CXCR3 expression leading to their accumulation in type 1 inflammatory sites (53, 54). Strategically generating Tregs that have low miR-17, miR-16a/16, or miR-142-3p or high miR-146a or let-7d expression may represent an approach to increase Treg suppressor function.

Four other miRNA, miR-7, miR-18a, miR-34a, and miR-155, have been shown to contribute to the stabilization of Treg suppressor function (47, 55, 56). It is suggested that the activity of these miRNA is mediated by Treg expression of SATB1, a genome organizer that regulates chromatin structure and gene expression (57). In mature Tregs, Foxp3 directly suppresses the *Satb1* locus. SATB1 is required for Treg suppressive function and to prevent the acquisition of helper T cell characteristics. Under non-activating conditions, Treg SATB1 is expressed at low levels; its forced expression results in complete loss of suppression to a level comparable with Foxp3 deficiency (55). In addition, T cell-specific deficiency of *Satb1* impaired Treg-super-enhancer activation and Treg signature gene expression in thymic Treg precursors, resulting in severe autoimmunity due to Treg deficiency (58). Foxp3 repression of SATB1 is achieved by inducing miRNA (miR-155, miR-21, miR-7, miR-34a, and miR-18a) binding to the *Satb1* 3′ untranslated region, indirectly suppressing SATB1. Thus, increased expression of these miRs may be particularly useful in inflammatory diseases such as GVHD by promoting Foxp3 expression and function.

### Effect of miRNA on Treg Fitness

MicroRNAs are instrumental in maintaining Treg fitness and survival. As mentioned earlier, miR-155 has proven functionally relevant to Treg fitness (a measure of survival/death and expansion) (59) with miR-155 KO mice having markedly reduced Treg number and impaired Treg proliferation (24, 60) associated with impaired STAT5 activation (24, 61) and increased SOCS1 (61). Conversely, miR-155 upregulation increases IL-2 sensitivity, promoting Treg fitness and proliferation (24, 59). miR-17–miR-92 also assists in maintaining Treg fitness (62). Treg-specific miR-17–miR-92 deletion increased Treg apoptosis and reduced proliferation, causing loss of Foxp3 expressing Treg in aged mice (63). By contrast, elevated miR-17–miR-92 in murine lymphocytes increased proliferation and reduced cell death (64), resulting in favored Treg accumulation in lymph nodes and non-lymphoid target tissues (63). By contrast, miR-146b impedes human Treg homeostasis (65), offering a potential therapeutic target that could be exploited to augment Treg fitness and survival. miR-146b antagomir treatment of Treg enhanced TRAF6 and the TRAF6–NF-κB–Foxp3 axis, resulting in improved Treg survival and proliferation (65). Thus, superior GVHD control may be acquired by increasing Treg fitness by upregulating miR-155 or reducing miR-17 or miR-146b expression.

### miRNA Effects on Genes That Contribute to Treg Suppressive Function

MicroRNA also regulates genes directly involved in Treg-mediated suppression. miR-15a/16 inhibits CTLA-4 expression (Figure 1C), precluding optimal Treg-mediated inhibition of dendritic cell (DC) maturation (48, 66). miR-142-3p inhibits expression of adenylyl cyclase 9, responsible for generating the inhibitory second messenger, cAMP (48, 66). Treg inhibition of Th1 proliferation and cytokine secretion *in vivo* has been demonstrated after transfer of exosomes containing suppressive miRNA, including let-7d, shown to be required for Treg-mediated amelioration of murine colitis (67).

## Concluding Remarks

The potential of miRNA therapeutics in human Treg cellular therapy was demonstrated by *ex vivo* inhibition of miR-146b that enhanced *in vitro* tTreg function and, upon adoptive tTreg transfer, superior *in vivo* xenogeneic GVHD lethality compared with scrambled miR control treatment (65). Clinical GVHD trials registered in http://clinicaltrials.gov employ *in vitro* generated iTreg and IL-10, TGFβ-producing CD4+ Foxp3− Treg type 1 (Tr1) cells (NCT01634217: MacMillan; NCT03198234: Roncarolo). Stability of Foxp3 expression and suppressive function are of paramount concern for Treg therapies in the intense GVHD inflammatory environment and would be amenable to miR manipulation. Similar Treg therapies are being used to treat diabetes (7) and could be used to restore normal expression of miR-342, miR-191, and miR-510 in Treg from diabetes patients (68). In addition, while outside the purview of this review, it should be noted that manipulating miRNA expression in other cell types (e.g., DCs) can have a significant impact on Treg induction and stability.

Regulatory T cell therapy, especially for GVHD, may benefit from miRNA therapeutics. First, because Tregs can be substantially expanded *in vitro*, permitting a higher Treg:Teffector ratio needed for optimal GVHD suppression and miRNA increases or knockdown can be tightly controlled. By treating just the cell of interest, the miR reagents needed are minimized. In contrast to autoimmunity and organ transplantation, acute GVHD typically is high risk in 1–3 months post-allo-HSCT until central and peripheral tolerance mechanisms become operative. Although clinical development of miRNA-based therapeutics has been slow, progress is being made. Locked nucleic acid-based miRNA and antagomirs prolong half-life, miRNA encapsulated in anionic nanoparticles greatly increases uptake, and antibodies can be incorporated into the nanoparticles to aid targeting, providing a new avenue for maximizing the efficacy and safety of Treg infusional therapies for GVHD (69–71).

## Author Contributions

KH, ML, and JN drafted the manuscript. KM prepared illustration. All the authors critically revised the manuscript for intellectual content and approved it for publication.

## Conflict of Interest Statement

The authors declare that the research was conducted in the absence of any commercial or financial relationships that could be construed as a potential conflict of interest.
